# Human Papillomavirus in the Lesions of the Oral Mucosa According to Topography

**DOI:** 10.1371/journal.pone.0069736

**Published:** 2013-07-29

**Authors:** Marinka Mravak-Stipetić, Ivan Sabol, Josip Kranjčić, Marjana Knežević, Magdalena Grce

**Affiliations:** 1 Department of Oral Medicine, School of Dental Medicine, University of Zagreb, Zagreb, Croatia; 2 Department of Molecular Medicine, Ruđer Bošković Institute, Zagreb, Croatia; University of Birmingham, United Kingdom

## Abstract

**Background:**

The association between human papillomavirus (HPV) types and oral lesions has been shown in many studies. Considering the significance that HPV has in the development of malignant and potentially malignant disorders of the oral mucosa, the purpose of this study was to investigate the prevalence of HPV DNA in different oral lesions. In addition, we wanted to elucidate whether the HPV infection is associated predominantly with either the lesion or a particular anatomic site of the oral cavity.

**Methodology/Principal Findings:**

The study included 246 subjects with different oral lesions, and 73 subjects with apparently healthy oral mucosa (controls). The oral lesions were classified according to their surface morphology and clinical diagnosis. The epithelial cells were collected with a cytobrush from different topographic sites in the oral cavity of the oral lesions and controls. The presence of HPV DNA was evaluated by consensus and type-specific primer-directed polymerase chain reaction. The HPV positivity was detected in 17.7% of oral lesions, significantly more than in apparently healthy mucosa (6.8%), with a higher presence in benign proliferative mucosal lesions (18.6%). High-risk HPV types were predominantly found in potentially malignant oral disorders (HPV16 in 4.3% and HPV31 in 3.4%), while benign proliferative lesions as well as healthy oral mucosa contained mainly undetermined HPV type (13.6 and 6.8%, respectively).

**Conclusions/Significance:**

The distribution of positive HPV findings on the oral mucosa seems to be more associated with a particular anatomical site than the diagnosis itself. Samples taken from the vermilion border, labial commissures, and hard palate were most often HPV positive. Thus, topography plays a role in HPV prevalence findings in oral lesions. Because of the higher prevalence of the high-risk HPV types in potentially malignant oral disorders, these lesions need to be continuously controlled and treated.

## Introduction

Oral lesions have different etiology, although most of them can be largely attributed to environmental exposures. Tobacco use, chewing areca nuts, and alcohol consumption are well-established risk factors of various tumors of the oral cavity [1]. Infectious agents also play an important role in the etiology of oral lesions [2]. For instance, human papillomaviruses (HPV) seem to be associated with a subset of oral benign proliferative and malignant lesions, the head and neck carcinoma, notably carcinoma of the oropharynx, tonsils and tongue [3]. Thus, HPV-positive oropharyngeal tumors are associated with oral sex, under 60 years of age, infrequent p53 gene mutation, and a more favorable clinical outcome, while HPV-negative carcinoma is associated with smoking, excessive alcohol use, above 60 years of age, frequent p53 gene mutation, and poor prognosis [4].

Mucosal (alpha genus) HPV types are known to infect both the genital and the upper respiratory tract [5]. Certain HPV types, notably types 16 and 18 causing primarily carcinoma of the cervix are recognized as human carcinogens, and are called high-risk (HR) types [6] In contrast, low-risk (LR) types, the most common being HPV types 6 and 11, primarily cause genital warts and laryngeal papillomas, and are rarely found in tumors [6]. High-risk HPV types can also cause other anogenital as well as upper aerodigestive tract carcinomas in women and men. Thus, HPV contributes to 47% oropharyngeal carcinomas and 11% other carcinomas of the oral cavity (tonsils and tongue), and HPV 16 is, as expected, the most commonly found type in oropharyngeal (90%) and oral carcinoma (96%) [7].

Earlier studies investigating the prevalence of HPV in oral carcinoma, potentially malignant disorders as well as in apparently healthy mucosa were inconsistent. Thus, the prevalence of HPV in normal oral mucosa, oral potentially malignant disorders and oral malignancies varies from 0 to 70%, 0 to 85%, and 0 to 100%, respectively (reviewed by Ha & Califano, 2004) [8]. The authors attributed those discrepancies to sampling variation and limitations of the molecular techniques used in different studies. Indeed, Kellokoski et al. (1992) showed that the same samples taken from healthy oral mucosa were positive for HPV in 3.8% by dot blot hybridization, while in 29.4% cases with polymerase chain reaction (PCR) [9]. In the recent meta-analysis, Syrjänen et al. (2011) pointed out that the sampling (oral scrapings, biopsies) is critical in the evaluation of HPV prevalence in oral specimens as well, and when the same sampling technique was used for both cases and controls, a strong association between HPV and oral potentially malignant disorders was found [10].

In this study we wanted to elucidate whether HPV infection is linked predominantly with either a particular disease or the site of infection in the mouth since this data is lacking. In addition, as there is no published data regarding HPV infection distribution in oral mucosa in Croatian population, the second aim of this study was to evaluate the prevalence of HPV infection and particular HPV types in different oral lesions in this population.

## Materials and Methods

### Study population

Patients with clinically distinct lesions of the oral mucosa referred to the Department of Oral medicine, School of Dental Medicine, University of Zagreb from 1995 to 2011, were included in this study. In total, there were 246 subjects with diagnosis, and 73 subjects with apparently healthy oral mucosa (controls). Lesions were initially diagnosed according to clinical features and confirmed by histopathologic analysis [11].

### Ethics Statement

Before the year 2001 verbal patient consent was obtained at the time of sample collection, and since then, written informed consent has been obtained from the participants. Both the relevant patient data (age, histopathological diagnosis, HPV testing results) and the DNA extracted from oral specimens were processed anonymously. HPV testing was done on demand through Laboratory service request forms, which had to be signed and stamped by the practicing dentist, and approved by The Rudjer Boskovic Institute of Zagreb. The whole study was approved by the Ethical Board of the School of Dental Medicine, University of Zagreb that is in line with the Helsinki declaration (DoH/Oct2008).

### Sample collection and processing

Scrapings of oral mucosa epithelial cells were taken with a cytobrush (Medscand AB, Sweden) from the site(s) of clinically visible lesion (coded according to the WHO oral topography chart) [12]. In control subjects (n = 73) only one sample was taken, which included several topographic locations (bilateral buccal, vestibular, retrocomissural, lingual, and sublingual mucosa), and was coded in this study as “whole oral mucosa”. In addition, 23 samples from widespread oral lesions (*stomatitis*, *lichen ruber planus*, and *lichen ruber erosivus*) were taken from several topographic locations and also nominated “whole oral mucosa” samples. The oral lesions were classified either by morphology, according to Nikitakis N.G. (2005) [13] or clinical diagnosis. Specifically, the premalignant oral lesions classified according to Warnakulasuriya S. et al. (2007) [14], were confirmed by histopathology [11].

Samples were collected in TES buffer (10 mM Tris-HCl; pH 7.5, 1 mM EDTA, pH 7.9; 0.5% SDS), frozen at −20°C, and transported to the laboratory for HPV detection and genotyping. DNA was isolated by the high salt method which allows the precipitation of proteins. Briefly, oral cell suspensions were treated with proteinase K (50 µg/ml) in TES buffer overnight at 37°C or 2 h at 56°C. Then, 1/3 volume of 5 M NaCl was added to the suspension, after 15–30 min incubation at 4°C, and 15 min centrifugation at 4°C, and 14,000 g, the supernatant was carefully collected, and treated with 2 volumes of 96% cold (−20°C) ethanol. The precipitation of DNA was facilitated by incubation at least 2 hour at −20°C, and 15 min at −70°C. The DNA was then collected after 15 min centrifugation at 4°C, and 14,000 g, one washing of excess salt, and a second short centrifugation, and resuspended in ultra pure sterile water (50–100 µl). The quality and quantity of DNA was determined spectrophotometrically.

### Human papillomavirus detection and typing

Detection and genotyping of alpha genus HPV types was done as described previously [15]. Briefly, in house polymerase chain reactions (PCR) were performed using MY09/11 or PGMY09/11 consensus PCR primers followed by type-specific PCR amplifying HPV types 6/11, 16, 18, 31, 33, 45, 52, and 58 in 4 separate multiplex reactions for all samples irrespective of the initial consensus PCR results. Negative or very weakly positive samples by MY/PGMY09/11 and type-specific PCR were further analyzed by L1C1/L1C2-1/L1C2-2, and/or GP5+/6+ consensus PCR. Human beta-globin was amplified together with the MY09/11 or PGMY09/11 consensus primers to verify the suitability of the isolated DNA for PCR. The PCR was performed with 5 ng/µl of DNA in each reaction and each run included positive and negative controls (all reaction components except DNA). All standard procedures to avoid DNA contamination were applied.

### Statistical data analysis

Patient data was recorded in the MS Access database and analyzed in MS Excel (Microsoft, USA). Chi square test and Chi square test for trend were used to determine statistically significant differences between sample groups and were calculated using Graph Pad Prism (GraphPad software, USA). P values <0.05 were considered statistically significant.

## Results

A total of 327 samples from 319 patients collected during the period from 1995 and 2011 were successfully analyzed for the presence of HPV DNA. In total, there were 204 lesions above oral mucosa surface (i.e. *keratosis*, *hyperplasia*), and 50 lesions of oral epithelium that affect only the tissue beneath the oral mucosa surface (i.e. erosions, ulcerations). In addition, there were 73 samples collected from patients without any clinically visible lesions or symptoms, and they served as a control group in this study. The patients were characterized by a total of 343 referring diagnosis descriptions. The majority of patients had only 1 given diagnosis (n = 300), 16 patients had 2 different diagnosis, while 3 patients each had 3, 4, or 5 different diagnosis throughout the study period, respectively. These clinical diagnoses were grouped into the 3 following categories: 1) potentially malignant disorders (*leukoplakia* [n = 63], *lichen ruben planus* [n = 39], *lichen ruber erosivus* [n = 8], and *eritroleukoplakia* [n = 6]); 2) benign proliferative lesions (*papilloma* [n = 38], *verruca vulgaris* [n = 21], *hyperkeratosis* [n = 12], *hyperplasia* [n = 15], *condyloma accuminata* [n = 1], and *leukoplakia villosa* [n = 1]); and 3) inflammatory lesions (*stomatitis* [n = 18], ulceration [n = 17], *glossitis* [n = 13], *chelitis* [n = 7], *reactio lichenoides* [n = 6], and *gingivitis* [n = 5]).

Table 1 presents the distribution of oral lesion by morphology and diagnosis according to gender and HPV positivity irrespective of topography. There were 203 women (average age 45.8±16.5 years, median 44) and 116 men (average age 41.5±18.0 years, median 38). Men had significantly more positive HPV lesions (27.1 *vs* 13.0%; p = 0.0096). The majority of HPV positive lesions in men were lesions above oral mucosa, while in women majority were beneath oral mucosa (31.4 *vs* 17.1%, respectively), but there were no statistical difference (p>0.05). In addition, men had more HPV positive potentially malignant disorders, in contrast to women who had more HPV positive benign proliferative lesions (32.4 *vs* 18.2%, respectively).

**Table 1:** Distribution of oral lesion by morphology and diagnosis according to gender and HPV positivity irrespective of topography.

Gender	Women						Men						Total	
HPV status	Negative		Positive		Subtotal		Negative		Positive		Subtotal			
	N	%	N	%	N	%	N	%	N	%	N	%	N	%
**Oral lesion morphology**														
**All lesion of the oral mucosa**	147	87.0	22	13.0	169	66.5	62	72.9	23	27.1	85	33.5	254	100
**Lesions above oral mucosa**	118	88.1	16	11.9	134	65.7	48	68.6	22	31.4	70	34.3	204	100
**Lesions beneath oral mucosa**	29	82.9	6	17.1	35	70.0	14	93.3	1	6.7	15	30.0	50	100
**Clinically healthy oral mucosa**	38	97.4	1	2.6	39	53.4	30	88.2	4	11.8	34	46.6	73	100
**Subtota**l	185	88.9	23	11.1	208	63.6	92	77.3	27	22.7	119	36.4	327	100
**Oral lesion diagnosis**														
**All diagnosis**	157	87.2	23	12.8	180	66.7	65	72.2	25	27.8	90	33.3	270	100
**Potential malignant disorders**	73	92.4	6	7.6	79	68.1	25	67.6	12	32.4	37	31.9	116	100
**Benign proliferative lesions**	45	81.8	10	18.2	55	62.5	23	69.7	10	30.3	33	37.5	88	100
**Inflammatory lesions**	39	84.8	7	15.2	46	69.7	17	85.0	3	15.0	20	30.3	66	100
**Normal mucosa (control)**	38	97.4	1	2.6	39	53.4	30	88.2	4	11.8	34	46.6	73	100
**Subtotal**	195	89.0	24	11.0	219	63.8	95	76.6	29	23.4	124	36.2	343	100

HPV was found statistically more often in a group of all clinically visible oral lesions (17.7%) *versus* the control group (6.8%; p = 0.023) with stronger association for lesions above oral mucosa surface (18.6%; p = 0.0171) (Table 2). There was no statistically significant difference between HPV positivity in lesions above *versus* below oral mucosa (p>0.05). As expected, HPV was found statistically more often in patients with any diagnosis (17.8%) *versus* the control samples (6.8%; p = 0.0219). Although there was a difference between potentially malignant disorders and controls in HPV positivity, as well as inflammatory lesions compared to controls, the difference did not reach statistical significance (15.5, and 15.2 vs 6.8%, respectively; p>0.05). HPV positivity was only significantly different between benign proliferative lesions *versus* the control samples (22.7% *vs* 6.8%, respectively; p = 0.0056).

**Table 2:** HPV genotypes distribution according to oral lesion morphology and diagnosis irrespective of topography.

HPV status	Total		Negative		Positive^a^		HPV 16		HPV 31		HPV 33		HPV 52		HPV 58		HPV 6/11		Multiple^b^		Unknown^c^	
	N	%	N	%	N	%	N	%	N	%	N	%	N	%	N	%	N	%	N	%	N	%
**Oral lesion morphology**																						
**All lesion of the oral mucosa**	254	100	209	82.3	45	17.7	6	2.4	8	3.1	1	0.4	1	0.4	1	0.4	4	1.6	4	1.6	20	7.9
**Lesions above oral mucosa**	204	100	166	81.4	38	18.6	6	2.9	6	2.9	1	0.5	1	0.5	1	0.5	4	2.0	3	1.5	16	7.8
**Lesions beneath oral mucosa**	50	100	43	86.0	7	14.0			2	4.0									1	2.0	4	8.0
**Clinically healthy oral mucosa**	73	100	68	93.2	5	6.8															5	6.8
**Subtotal**	327	100	277	84.7	50	15.3	6	1.8	8	2.4	1	0.3	1	0.3	1	0.3	4	1.2	4	1.2	25	7.6
**Oral lesion diagnosis**																						
**All diagnosis**	270	100	222	82.2	48	17.8	6	2.2	8	3.0	1	0.4	1	0.4	1	0.4	4	1.5	4	1.5	23	8.5
**Potential malignant disorders**	116	100	98	84.5	18	15.5	5	4.3	4	3.4	1	0.9	1	0.9	1	0.9	1	0.9	1	0.9	4	3.4
**Benign proliferative lesions**	88	100	68	77.3	20	22.7	1	1.1	2	2.3							3	3.4	2	2.3	12	13.6
**Inflammatory lesions**	66	100	56	84.8	10	15.2			2	3.0									1	1.5	7	10.6
**Normal mucosa (control)**	73	100	68	93.2	5	6.8															5	6.8
**Subtotal**	343	100	290	84.5	53	15.5	6	1.7	8	2.3	1	0.3	1	0.3	1	0.3	4	1.2	4	1.2	28	8.2

a HPV types 18 and 45 were not determined in any case.

b three cases with HPV 6/11 and HPV 16, and one case with HPV 6/11 and HPV 31.

c HPV X.

The overall HPV presence in samples of groups with different clinical diagnosis is shown in Table 2. The determination of HPV types 6/11, 16, 18, 31, 33, 45, 52, and 58 was successful in 50% HPV-positive samples, and the other half remained untyped (HPV X). As expected, most high-risk HPV types were found in potentially malignant disorders, while in benign proliferative lesions more HPV types X were detected. Apart of HPV X, high-risk HPV 16 and HPV 31 were the most commonly found in lesions above oral mucosa in 2.9% cases each, and particularly in potentially malignant disorders in 4.3 and 3.4%, respectively. In clinically apparently healthy mucosa HPV positivity was 6.8%, and in all cases the type was not determined (HPV X). Undetermined HPV types were also common in benign proliferative lesions and inflammatory lesions, found in 13.6 and 10.6%, respectively, while less in potentially malignant disorders (3.4%).

**Figure 1:**
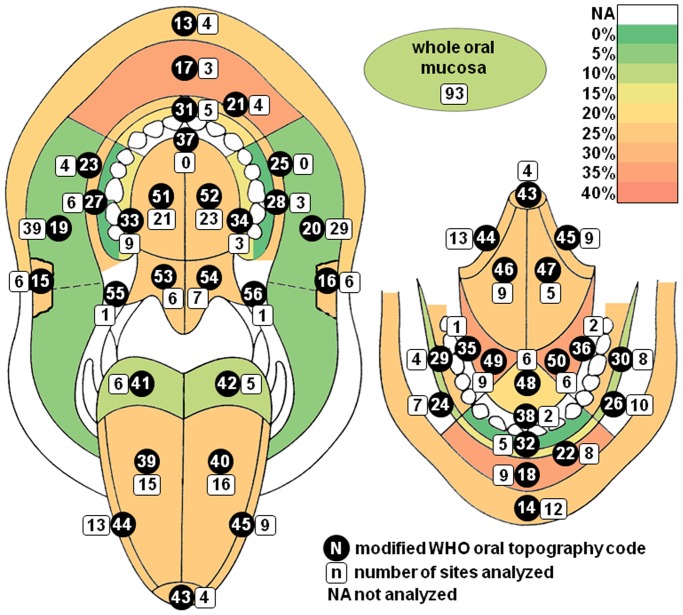
Frequency of HPV detection at different regions of the oral mucosa. The topography of the oral mucosa is coded according to the modified WHO oral topography codes by Roed-Petersen and Roenstrup [12], [22]: vermilion border - upper (13), lower (14), labial commissures - right (15), left (16), labial mucosa - upper (17), lower (18), labial sulci - upper (21), lower (22), cheek (bucal mucosa) - right (19), left (20), buccal sulcus - right upper (23) lower (24), buccal sulcus – left upper (25) lower (26), upper gingiva or edentulous alveolar ridge buccally - right (27), left (28), lower gingiva or edentulous alveolar ridge - right (29), left (30), upper anterior gingiva and edentulous ridge labially (31), lower anterior gingiva or edentulous ridge labially (32), upper posterior gingiva or edentulous alveolar ridge palatally - right (33), left (34), lower posterior gingiva or edentulous alveolar ridge lingually - right (35), left (36), anterior gingiva or edentulous ridge palatally (37), and lingually (38), dorsum of the tongue - right (39), left (40), base of the tongue - right (41), left (42), tip of the tongue (43), margine of the tongue - right (44), left (45), surface of the tongue - right (46), left (47), frontal floor of mouth (48), lateral floor of mouth - right (49), left (50), hard palate - right (51), left (52), soft palate - right (53), left (54), anterior tonsillar pillar - right (55), left (56); color scale indicates frequency of HPV positivity in the respective locations; for regions with less than 3 samples analysed the frequency was not calculated; symetrical regions were counted as one for the analysis.

Each analyzed sample had accompanying sampling location information according to the modified WHO oral topography coding [12]. Oral scrapings were taken from a single location in 34.5% cases, from 2 mostly adjacent locations in 29.5%, and more than 2 locations in 7.4%. Scrapings of the “whole oral mucosa” were taken in 28.6% cases, mostly in case of clinically healthy oral mucosa. Figure 1 graphically represents the distribution and frequency of HPV positivity at different topographical regions of the oral cavity.

Because of the limited number of subgroups of a particular diagnosis-location combination we have grouped data according to HPV frequency. For that we calculated the median and the standard deviation of the total HPV frequency by location or diagnosis, respectively. Moderate HPV frequency group included the median and ±1/2 standard deviation. High frequency and low frequency groups were above and below the moderate group, respectively. The resulting findings are summarized in Table 3. The Chi square test for trend was used on row-column subtotal intersections within each subgroup of diagnosis (low, moderate, high) to assess the influence of topography on disease subgroup. The influence of diagnosis within each subgroup of topography (low, moderate, high) was also assessed with the same test. The statistical analysis indicated that HPV positivity significantly correlates with the location in moderate and high HPV frequency -diagnosis subgroups (p = 0.041 and p = 0.038, respectively), while for low HPV frequency diagnosis group the p value was not significant (p = 0.366). On the other hand, the diagnosis was significantly affecting only the moderate HPV frequency topography subgroup (p = 0.003), while it was not so for the high and low frequency topography subgroups, p = 0.111 and p = 0.054, respectively. In summary, HPV positivity seems to be more associated to particular location than diagnosis itself.

**Table 3:** The frequency of HPV positivity in different combinations of clinical diagnosis and topography of lesions.

N HPV+/N total (%)^a^	Buccal mucosa	Posterior gingiva	Whole mucosa	Subtotal	Anterior gingiva	Labial and buccal sulci	Ventral tongue mucosa and floor of mouth	Tongue	Subtotal	Labial commissures	Labial mucosa	Palate	Vermilion border	Subtotal	Total^b^	HPV frequency
**Fibroma**		0/1 (0)	0/1 (0)	0/2 (0)		0/1 (0)		3/3 (100)	3/4 (75)		0/1 (0)	0/2 (0)	1/1 (100)	1/4 (25)	4/10 (40)	
**Papilloma**	1/5 (20)	0/2 (0)	3/5 (60)	4/12 (33.3)	1/2 (50)	1/6 (16.7)		1/9 (11.1)	3/17 (17.6)	2/2 (100)	3/7 (42.9)	3/12 (25)	2/5 (40)	10/26 (38.5)	17/55 (30.9)	
**Aphtae**	1/3 (33.3)	0/1 (0)	0/1 (0)	1/5 (20)	0/1 (0)	0/1 (0)	1/3 (33.3)	1/1 (100)	2/6 (33.3)	0/2 (0)	1/2 (50)	0/1 (0)	1/1 (100)	2/6 (33.3)	5/17 (29.4)	
**Stomatitis**	0/3 (0)		2/2 (100)	2/5 (40)			0/2 (0)	0/5 (0)	0/7 (0)	0/1 (0)	0/1 (0)	4/11 (36.4)	0/1 (0)	4/14 (28.6)	6/26 (23.1)	
**Leukoplakia**	0/7 (0)	1/9 (11.1)	0/2 (0)	1/18 (5.6)	3/15 (20)	4/16 (25)	2/13 (15.4)	2/11 (18.2)	11/55 (20)	1/4 (25)	0/4 (0)	5/13 (38.5)	2/7 (28.6)	8/28 (28.6)	20/101 (19.8)	
**Lichen ruber erosivus**	0/6 (0)		0/1 (0)	0/7 (0)				2/4 (50)	2/4 (50)						2/11 (18.2)	
**Subtotal**	2/24 (8.3)	1/13 (7.7)	5/12 (41.7)	8/49 (16.3)	4/18 (22.2)	5/24 (20.8)	3/18 (16.7)	9/33 (27.3)	21/93 (22.6)	3/9 (33.3)	4/15 (26.7)	12/39 (30.8)	6/15 (40)	25/78 (32.1)	54/220 (24.5)	High (≥18.2%) p = 0.038
**Hyperplasia**	0/4 (0)			0/4 (0)	0/1 (0)	0/1 (0)		0/2 (0)	0/4 (0)			2/3 (66.7)	0/1 (0)	2/4 (50)	2/12 (16.7)	
**Chelitis**	0/3 (0)			0/3 (0)									1/5 (20)	1/5 (20)	1/8 (12.5)	
**Verruca**	0/1 (0)		1/2 (50)	1/3 (33.3)	0/3 (0)	0/1 (0)		0/8 (0)	0/12 (0)	0/2 (0)	0/2 (0)	1/6 (16.7)	1/2 (50)	2/12 (16.7)	3/27 (11.1)	
**Lichen ruber planus**	2/33 (6.1)	0/1 (0)	0/3 (0)	2/37 (5.4)	1/2 (50)	0/2 (0)		1/6 (16.7)	2/10 (20)	1/4 (25)		1/6 (16.7)		2/10 (20)	6/57 (10.5)	
**Glositis**			0/1 (0)	0/1 (0)	0/2 (0)			2/17 (11.8)	2/19 (10.5)	0/0 (0)					2/20 (10)	
**Other diseases**	0/4 (0)	1/4 (25)	0/3 (0)	1/11 (9.1)	0/11 (0)	0/3 (0)		0/1 (0)	0/15 (0)	0/0 (0)		2/8 (25)	0/1 (0)	2/9 (22.2)	3/35 (8.6)	
**Subtotal**	2/45 (4.4)	1/5 (20)	1/9 (11.1)	4/59 (6.8)	1/19 (5.3)	0/7 (0)		3/34 (8.8)	4/60 (6.7)	1/6 (16.7)		6/23 (26.1)	2/9 (22.2)	9/40 (22.5)	17/159 (10.7)	Moderate (6.8–18.2%) p = 0.041
**No disease**			5/73 (6.8)	5/73 (6.8)											5/73 (6.8)	
**Keratosis**	0/3 (0)		0/1 (0)	0/4 (0)	0/1 (0)	0/3 (0)		0/3 (0)	0/7 (0)	0/2 (0)		0/3 (0)	0/2 (0)	0/7 (0)	0/18 (0)	
**Reactio lichenoides**	0/8 (0)			0/8 (0)		0/2 (0)			0/2 (0)			0/1 (0)		0/1 (0)	0/11 (0)	
**Subtotal**	0/11 (0)		5/74 (6.8)	5/85 (5.9)	0/1 (0)	0/5 (0)		0/3 (0)	0/9 (0)	0/2 (0)		0/4 (0)	0/2 (0)	0/8 (0)	5/102 (4.9)	Low (≤6.8%) p = 0.336
**Total**	4/80 (5)	2/18 (11.1)	11/95 (11.6)	17/193 (8.8)	5/38 (13.2)	5/36 (13.9)	3/18 (16.7)	12/70 (17.1)	25/162 (15.4)	4/17 (23.5)	4/17 (23.5)	18/66 (27.3)	8/26 (30.8)	34/126 (27)	76/481 (15.8)	
**HPV frequency**	Low (≤12.8%) p = 0.054				Moderate (12.8–20.6%) p = 0.003					High (≥20.6%) p = 0.111						

a Data is presented as number of HPV positive samples/total number of samples at the intersection of row and column (percentage within subgroup), empty cells contain no samples i.e. 0/0(0);

b There were samples from multiple locations and/or diagnosis and such samples were counted in each respective row or column. However, samples described with multiple topographical codes of the same region (i.e. tongue codes are 39 to 45) were counted only once within that subgroup. Thus the number of diagnosis-location pairs is greater than the number of individual samples in the study.

## Discussion

This study is the only study on HPV prevalence in oral mucosal lesions in the Croatian population. Although the collection of the study population took 16 years, the advantage of this study is its sample size and uniformity of material, sampling, and further processing procedures. All the oral scrapings were taken in the single clinic under the same conditions, and DNA preparation and HPV analysis was also done in the same manner in the virological laboratory. Clinical diagnosis of the oral lesions and sampling was evaluated by a single specialist. In each case, oral epithelial cells were taken with the cytobrush and biopsies were excluded from this study. In addition, each specimen was classified according to the WHO topography code [12] in order to evaluate the relationship of HPV infection and oral lesions at the different locations of the mouth.

Three sets of consensus primers were used to maximize the identification of alpha-HPV DNA. In addition, genotyping for specific HPV types was performed on all samples regardless of consensus PCR result to further increase the HPV detection sensitivity. Types selected for genotyping were chosen due to their abundance in cervical carcinoma in the world [16]. Similar studies of HPV detection in oral cells by PCR were done earlier confirming the significant association of HPV positivity in oral carcinoma, potentially malignant disorders such as oral *leukoplakia*, oral *lichen ruber planus*, and epithelial *dysplasia* (reviewed in Syrjänen et al., 2011) [10]. This study includes all the listed oral lesions except oral carcinoma.

The HPV prevalence in healthy oral mucosa [17] and in oral lesions [18] is higher in men than women both in our study and the literature. However, regarding the different morphology and diagnosis, herein no statistically significant difference was found because of the low number of study subgroups.

As expected, the lesions with morphological changes above the oral mucosa and benign proliferative lesions contained HPV most often. Control samples of clinically healthy oral mucosa were HPV positive in 6.8% cases, but contained only HPV X. The comparable findings were found in recent systematic review by Kreimer et al. (2010) [19] where they found 4.5% overall HPV positivity in healthy subjects, of which 1.3% had HPV 16.

In this study, half of the detected HPV types with consensus primers remained untyped (HPV X). They can represent other alpha-HPV types since approximately 40 types infect the anogenital and oral mucosa and in this study only the most common were genotyped [6]. In addition, beta-HPV and gamma-HPV, which mostly infect skin and are considered to be cutaneous types, may also be present in the oral cavity and can be detected with (PG)MY09/11 [20], and probably with other consensus primers as well. The frequency of HPV X was the lowest in potentially malignant oral disorders, which was expected since these lesions are most often caused by the most common high-risk HPV types and those types were genotyped herein. However, it appears that oral benign proliferative lesions more often contain either less common high-risk types or other HPV types and thus remained untyped in this study. Such lesions are therefore less likely to progress into carcinoma.

Our results showed that topography plays a role in HPV prevalence in oral lesions. Even though the same lesions were found in different regions of the oral cavity, the HPV positivity was higher in specific topographical regions irrespective of diagnosis. It was not possible to analyze different diagnosis-topography combinations due to small sample subgroups, thus the data were grouped according to HPV frequency associated with the diagnosis and topography of lesion (Table 3). The statistical analysis has shown that HPV positivity of the lesions that belong to the moderate or high HPV frequency-diagnosis subgroups is significantly associated with location. The topography was not significantly associated with low HPV frequency-diagnosis subgroup as the number of HPV positive samples was very small, but it was significantly associated with the moderate HPV frequency topography subgroup. Undoubtedly, it is true that some particular diagnosis are more associated with HPV and thus more often HPV positive, however, these data show that topography of the lesion is significantly associated to the HPV positivity of a particular lesion, and even somewhat more than the diagnosis. One of the possible explanations for the observed data is that the locations with the highest HPV positivity (*vermilion border*, hard palate, *labial mucosa* and *labial commissures*) are also the most frequently exposed sites to microtrauma that is prerequisite for HPV infection transmission.

In our study, *papillomas* could be found throughout the oral mucosa except ventral tongue and floor of the mouth. As papillomas are mostly caused by HPV infection this finding is in line with the view that HPV infection of the mouth is multifocal [21]. In addition, *leukoplakia* was also present on all anatomic sites and the most frequently on the tongue mucosa. In contrast, *oral lichen planus* was found predominatly on buccal mucosa but with the least HPV positivity, which is associated with the role of local cellular immunity in its immunopathogenesis [22].

Although HPV infection is associated with potentially malignant oral disorders it is not possible to predict the likelihood of an HPV infection from the clinical features of these lesions [23]. Therefore, it is important to know the topographic distribution of the HPV infection, in other words, its affinity for particular anatomic sites on oral mucosa, to be able to determine the risk and then follow-up these regions more closely. Our study in this context significantly contributes to better understanding of the field.

Our study is among the largest studies on oral HPV prevalence published so far. From our findings it appears that HPV prevalence in oral cavity in Croatian population is comparable to nearby Italian population both for potentially malignant lesions and normal mucosa [23], however our overall HPV prevalence is on the lower end of reported oral HPV prevalence from other studies.

One of the important findings of this study can partially elucidate the large discrepancy of observed HPV prevalence in previous studies as we have shown that in addition to the association of HPV with some particular diagnosis, HPV is also associated to individual anatomic sites or regions of the oral mucosa. Thus, previous studies analyzing samples of a particular diagnosis taken from different regions of oral cavity reached different conclusions. Even though topographical coding was available for a long time, very few studies, if any, noted the sampling locations and this should be changed in the future to make studies more comparable and reported HPV prevalence more reliable.
